# An open science resource for accelerating scalable digital health research in autism and other neurodevelopmental conditions

**DOI:** 10.1038/s41593-025-02146-3

**Published:** 2025-12-30

**Authors:** Micha Hacohen, Adam Levy, Hadas Kaiser, LeeAnne Green Snyder, Alpha Amatya, Brigitta B. Gundersen, John E. Spiro, Ilan Dinstein

**Affiliations:** 1 Psychology Department, Ben Gurion University of the Negev, Beer Sheva, Israel; 2 Azrieli National Centre for Autism and Neurodevelopment Research, Ben Gurion University of the Negev, Beer Sheva, Israel; 3 Simons Foundation, New York, NY USA

**Keywords:** Diagnostic markers, Sleep, Autism spectrum disorders, Psychology

## Abstract

The Simons Sleep Project (SSP) is an open-science resource designed to accelerate digital health research into sleep and daily behaviors of autistic children. The SSP contains data from Dreem3 EEG headbands, multi-sensor EmbracePlus smartwatches and Withings’ sleep mats, as well as parent questionnaires and daily sleep diaries. It contains data from >3,600 days and nights collected from 102 children (aged 10–17 years) with idiopathic autism and 98 of their nonautistic siblings, and enables access to whole-exome sequencing for all participants. Here we present the breadth of available harmonized data and show that digital devices have higher accuracy and reliability compared to parent reports. The data show that autistic children have longer sleep-onset latencies than their siblings and longer latencies are associated with behavioral difficulties in all participants, regardless of diagnosis. The results highlight the advantages of using digital devices and demonstrate the opportunities afforded by the SSP to study autism and develop broad digital phenotyping techniques.

## Abstract (web summary)

This paper describes the Simons Sleep Project, an open resource designed to accelerate research into the sleep and daily behaviors of autistic children using synchronized recordings from multiple wearable and nearable devices for >3,600 days and nights.

## Main

The use of wearable or nearable devices with activity, sleep and other health-monitoring applications has grown rapidly over the last decade^1^. These devices offer exciting opportunities for research in many scientific fields because they enable objective and direct quantification of behavior and physiology from large cohorts of participants in ecological settings (that is, during daily life). The analysis of data from these devices requires the development of automated algorithms that can identify and quantify specific behaviors and physiological events of interest (for example, tantrums, physical exercise, epileptic seizures and sleep)^2–5^. To develop accurate, reliable and transparent algorithms, the scientific community needs access to shared open resources with raw data from simultaneous recordings of multiple devices. Such developments will ultimately be valuable, for example, for performing large longitudinal natural history studies, revealing genotype–phenotype relationships and developing objective clinical outcome measures.

The goal of the Simons Sleep Project (SSP) is to create a resource for developing such techniques within the context of autism research and an initial focus on studying sleep behavior and neurophysiology. Autism is a behavioral diagnosis that is based on the presence of two core symptom clusters, social communication difficulties and restricted and repetitive behaviors^6^. The autism population is, however, highly heterogeneous and most individuals exhibit one or more co-occurring symptoms, which include language delays, intellectual disability, challenging behaviors (for example, tantrums), sensory problems, hyperactivity, sleep disturbances and a wide variety of mental health problems (for example, depression and anxiety)^7,8^. These additional symptoms can be equally or more debilitating for individuals with autism and their families than the core symptoms. Currently available wearable or nearable devices will be particularly useful for quantifying the severity of co-occurring symptoms, studying their etiology and underlying mechanisms, characterizing their relationship with core autism symptoms and developing new techniques for prevention and intervention. Insights from studying autism should be applicable to a wide variety of neurodevelopmental, psychiatric and neurological disorders with overlapping symptoms.

Sleep disturbances are one common co-occurring symptom in autism, with previous questionnaire-based studies reporting that >50% of individuals with autism have severe sleep disturbances^9,10^, in contrast to ~20% of typically developing individuals^11^. The most frequently reported sleep disturbances include difficulties falling asleep, frequent awakening throughout the night and shorter overall sleep duration^12^. Studies have reported that the severity of sleep disturbances is weakly correlated with the severity of cognitive difficulties and core autistic spectrum disorder symptoms^13^ and moderately correlated with sensory problems and challenging behaviors^14–16^. These studies suggest that, in some individuals with autism, sleep disturbances may exacerbate the severity of other symptoms, including core symptoms, which may also be generated by shared underlying neurophysiology. In rodents, sleep deprivation during early development has been shown to generate autism-like behaviors^17,18^ that, in some cases, may be ameliorated by improving sleep^19^. In children with autism, treatment with melatonin has been shown to improve sleep disturbances and ameliorate challenging behaviors and additional symptoms^20^. A deeper understanding of specific sleep disturbances and their underlying neurophysiology has the potential to reveal important mechanistic and clinical insights for autism and child development more broadly.

An important caveat of the human studies described above is that they primarily used questionnaires to assess sleep disturbances, thereby basing their conclusions on subjective and potentially biased measures of sleep reported by the participants or their caregivers. To address this, several studies have used overnight polysomnography (PSG) recordings to measure sleep directly and objectively from individuals with autism. During PSG recordings, participants are connected to multiple electroencephalogram (EEG), electro-oculogram and electromyogram electrodes that record neural activity, enabling direct identification of sleep and wake periods as well as sleep architecture (that is, sleep staging) and neurophysiology (for example, sleep spindles). Most PSG studies have reported that individuals with autism exhibit significantly shorter total sleep time (TST) than controls but do not exhibit differences in wake-after-sleep onset (WASO)^21–24^. This suggests that some, but not all, parent-reported sleep disturbances are apparent in direct measures of sleep. These studies also reported diverse findings regarding sleep neurophysiology, including less rapid eye movement sleep^23^, fewer sleep spindles^25,26^ and weaker slow-wave activity^24^ in participants with autism relative to controls. Although PSG studies are considered the ‘gold standard’ for studying sleep, it is important to consider that all PSG studies described above recorded sleep during a single night while participants were connected to bulky equipment as they slept in a sleep laboratory. As sleep varies considerably across nights^27^, and individuals with autism often find changes in routine particularly aversive^28^, a single PSG recording in a sleep clinic may not faithfully represent their typical sleep behavior at home (that is, low ecological validity). PSG research can be expanded to the home environment using EEG headbands such as the Dreem3 (Beacon Inc.), which enables remote recordings with multiple EEG channels. Several recent studies of patients with Alzheimer’s disease^29^ and those with 22.q11.2 deletion syndrome^30^ have demonstrated that these data can be collected over multiple nights and yield high-quality EEG data for examining sleep spindles and performing spectral analyses. Such devices have not, however, been used to date in autism research.

To objectively estimate sleep in autism within the home environment, additional studies have used Actigraphy devices, which estimate sleep from participant movements. A recent meta-analysis of such studies concluded that sleep-onset latency (SOL) was significantly longer (by 12 min) and TST significantly shorter (by 15 min) in recorded individuals with autism relative to controls whereas WASO did not differ across groups^2^. As with PSG findings, these results suggest that some, but not all, parent-reported sleep disturbances are apparent in direct measures of sleep. Note that, unlike PSG studies, Actigraphy studies estimate sleep and wake periods indirectly from participant movements and several studies have demonstrated that these estimates are inaccurate relative to PSG measures, particularly when quantifying SOL^31^ and WASO^32^. This has motivated the introduction of more modern multi-sensor smartwatches that include photoplethysmography (PPG) sensors for measuring heart rate (HR) and HR variability in addition to measuring movements with accelerometry. The added information from PPG sensors seems to enable higher accuracy in identifying sleep or wake segments and accurately quantifying WASO^33^, but such devices have not been used in autism research to date. Additional nearable devices such as the Withings’ sleep mat, which is placed under the mattress and senses pressure changes, can provide information about time in bed and participant movements that could further increase the accuracy of sleep measures at home^34^. Such devices have also not been used in autism research to date.

The SSP is intended to rapidly accelerate research in autism and other psychiatric disorders by providing the research community with an open data resource that contains raw and processed data from >3,600 nights that were recorded with three devices: Dreem3 EEG headband (Beacon Inc.), EmbracePlus smartwatch with accelerometer, PPG, electrodermal activity (EDA) and skin temperature sensors (Empatica Inc.) and Withings’ sleep mat (Withings Inc.). Corresponding parent-reported data were also collected from a daily sleep diary and baseline questionnaires. The EmbracePlus and Dreem3 devices were selected for their ability to record and save raw sensor-level data, which is critical for performing transparent and reliable research which is not based on data from proprietary algorithms that are closed source and can be changed by device manufacturers without notice.

We recorded data from 102 autistic children and 98 of their nonautistic siblings (aged 10–17 years) who live in the same household, thereby constraining multiple familial factors, which are likely to have a strong impact on the children’s sleep environment and sleep hygiene^35^. Note that siblings of autistic children are known to exhibit a considerably higher prevalence of neurodevelopmental and psychiatric conditions relative to the general population^36^, likely because they share 50% of their sibling’s genes. For example, compared to the general population, siblings have a 3.5-fold higher prevalence of attention deficit hyperactivity disorder (ADHD)^37^, which has also been associated with increased sleep disturbances^38^. The SSP should, therefore, be particularly useful for researchers using dimensional^39^ or transdiagnostic^40^ approaches to disentangle the relationships between specific sleep disturbances and specific neurodevelopmental symptom domains, some of which are shared across siblings and others that are not.

The availability of parallel recordings from multiple devices or sensors, as well as inclusion of subjective and objective sleep measures, offers a unique opportunity to compare measures of sleep behavior and neurophysiology, assess the agreement between objective and subjective sleep measures and study sleep disturbances in autistic children and their siblings at a level of detail that has not been possible to date. Moreover, whole-exome sequencing data are also available for all participants, enabling potential exploration of genotype–phenotype relationships. The goal of this report is to introduce the SSP dataset, demonstrate its potential and facilitate its utilization by the broad research community.

## Results

**Fig. 1:**
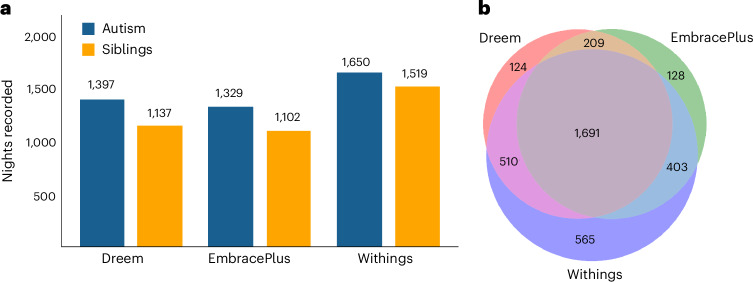
Data overview. **a**, Summary of nights and days recorded with Dreem, EmbracePlus and Withings’ devices from the autism (blue) and siblings (yellow) groups. **b**, Venn diagram presenting the number of nights recorded with each device and their overlap (that is, simultaneous recordings with multiple devices).

Data were successfully recorded from 102 autistic children (86 boys) and 98 nonautistic siblings (53 boys), between the ages of 10 and 17 years, from 102 families in the Simons Powering Autism Research for Knowledge (SPARK)^41^ cohort (Fig. 1). Children were recorded in their home setting over multiple days (EmbracePlus) and nights (all devices), with the final dataset comprising 3,630 nights: 1,691 nights recorded with all three devices, 1,122 nights recorded with two devices and 817 nights recorded with a single device (8,134 device recordings in total). Corresponding sleep diary data are also available for 2,630 of these nights. Note that the counts above refer to Dreem, EmbracePlus and Withings night-time recordings with at least 3 h of sleep (Methods).

Parents of all SSP participants completed baseline questionnaires regarding each child’s behavioral abilities and difficulties (Table 1). There were no significant correlations between the amount of data (that is, number of recorded nights) from individual autistic children per device and the children’s behavioral abilities or difficulties. This was the case for Children’s Sleep Habits Questionnaire (CSHQ) total scores (*r*(102) > −0.17, *P* > 0.09), Child Behavior Checklist (CBCL) total (*r*(102) > −0.13, *P* > 0.18), CBCL *Diagnostic and Statistical Manual of Mental Disorders*, 5th edn (DSM-5)^6^ ADHD (*r*(102) > −0.07, *P* > 0.5), CBCL DSM-5 anxiety (*r*(102) > −0.16, *P* > 0.11) and CBCL DSM-5 depression (*r*(102) > −0.14, *P* > 0.17) scores. Similarly, there were no significant correlations with Social Responsiveness Scale, 2nd edn (SRS-2) total scores (*r*(102) > −0.08, *P* > 0.42) or Vineland Adaptive Behavior Composite (*r*(102) < 0.12, *P* > 0.23), Vineland communication (*r*(102) < 0.16, *P* > 0.1), Vineland daily living skills (*r*(102) < 0.1, *P* > 0.31) or Vineland social (*r*(102) < 0.14, *P* > 0.17) scores. There were marginally significant correlations between Repetitive Behavior Scale—Revised (RBS-R) scores and the number of nights recorded with Dreem or Empatica (*r*(102) < −0.21, *P* < 0.037, not significant after Bonferroni’s correction), but not with Withings (*r*(102) > −0.1, *P* = 0.3). Taken together, these findings show that data were collected with all three devices from children with a wide variety of behavioral abilities and difficulties in an unbiased manner.

**Table 1:** Participant characteristics

Variable	Autism (*n* = 102)	Siblings (*n* = 98)	Statistics
**Age, mean years** **±** **s.d.**	13.94 ± 2.13	13.46 ± 2.16	*t* = 1.56, *P* = 0.120
**Sex** (male/female)	86/16	52/46	***x***^**2**^ **=** **21.34,** ***P*** = **0.000**
**SRS-2 total, mean** **±** **s.d.**	73.71 ± 10.01	47.89 ± 7.14	***t*** = **20.83,** ***P*** = **0.000**
**RBS-R total, mean** **±** **s.d.**	24.52 ± 14.05	4.15 ± 6.76	***t*** = **12.91,** ***P*** = **0.000**
**Vineland, mean** **±** **s.d.**			
Adaptive Behavior Composite	77.64 ± 12.81	102.36 ± 14.26	***t*** = **−12.84,** ***P*** = **0.000**
Communication	82.17 ± 14.02	104.50 ± 10.55	***t*** = **−12.63,** ***P*** = **0.000**
Socialization	72.15 ± 18.33	101.09 ± 12.43	***t*** = **−12.95,** ***P*** = **0.000**
Daily living skills	82.05 ± 16.02	101.22 ± 16.47	***t*** = **−8.30,** ***P*** = **0.000**
**CBCL total, mean** **±** **s.d.**	52.97 ± 9.82	47.31 ± 10.96	***t*** = **3.83,** ***P*** = **0.000**
**ABC, mean** **±** **s.d.**			
Irritability	8.24 ± 7.29	3.46 ± 5.02	***t*** = **5.35,** ***P*** = **0.000**
Social withdrawal	9.42 ± 7.84	2.68 ± 4.18	***t*** = **7.50,** ***P*** = **0.000**
Stereotypical behavior	4.39 ± 3.93	0.34 ± 1.19	***t*** = **9.74,** ***P*** = **0.000**
Hyperactivity	11.44 ± 7.99	4.64 ± 6.85	***t*** = **6.42,** ***P*** = **0.000**
Inappropriate speech	2.84 ± 2.62	0.61 ± 1.11	***t*** = **7.74,** ***P*** = **0.000**
**CSHQ total, mean** **±** **s.d.**	46.19 ± 8.05	43.31 ± 6.67	***t*** = **2.74,** ***P*** = **0.007**
**Sensory Profile 2, mean** **±** **s.d.**			
Sensory sensitivity	0.86 ± 0.91	−0.56 ± 0.94	***t*** = **10.86,** ***P*** = **0.000**
Sensory registration	0.88 ± 0.92	−0.56 ± 0.99	***t*** = **10.62,** ***P*** = **0.000**
Sensory avoidance	0.91 ± 0.86	−0.55 ± 0.88	***t*** = **11.79,** ***P*** = **0.000**
Sensory seeking	0.12 ± 0.72	−0.91 ± 0.83	***t*** = **9.29,** ***P*** = **0.000**
**Additional diagnoses, no. (%)**			
ADHD	58 (56.86)	30 (29.41)	***x***^2^ = **12.93,** ***P*** = **0.000**
Depression	33 (32.35)	23 (23.47)	***x***^2^ = 1.54, *P* = 0.21
Anxiety	16 (15.68)	11 (11.22)	***x***^2^ = 0.97, *P* = 0.32
**Medication, no. (%)**			
Any medication	66 (69.47)	49 (52.69)	***x***^**2**^ **=4** **.890,** ***P*** = **0.027**
Antidepressants	31 (32.63)	13 (13.98)	***x***^**2**^ = **8.110,** ***P*** = **0.004**
Stimulants	30 (31.58)	13 (13.98)	***x***^**2**^ = **7.285,** ***P*** = **0.007**
Antianxiety	26 (27.37)	13 (13.98)	***x***^**2**^ = **4.343,** ***P*** = **0.037**
Antipsychotics	12 (12.63)	1 (1.08)	***x***^**2**^ = **8.038,** ***P*** = **0.005**
Sleep aids	8 (8.42)	8 (8.6)	***x***^2^ = 0, *P* = 1
Anticonvulsants	4 (4.21)	2 (2.15)	***x***^2^ = 0.151, *P* = 0.698

Mean, s.d., number (*n*) and percentage are reported per measure as relevant. Questionnaires included SRS-2, RBS-R, Vineland Adaptive Behavior Scale (Vineland), CBCL, Aberrant Behavior Checklist (ABC), CSHQ, Family Inventory of Sleep Habits (FISH) and Sensory Profile, 2nd edn. Bold indicates significant differences across groups.

### Comparison between the SSP sample and the entire SPARK cohort

Some of the questionnaires completed by parents of SSP autistic children, including the SRS, RBS-R, CBCL and Vineland, were also available for other children in SPARK, enabling us to compare our SSP sample with thousands of autistic children aged 10–17 years in the SPARK cohort (while excluding SSP children from the SPARK group). A series of Student’s *t*-tests revealed that SPARK children exhibited significantly higher SRS (*t*(103.73) = 3.45, *P* < 0.001, Cohen’s *d* = 0.33) and RBS-R (*t*(103.34) = 5.76, *P* < 0.001, Cohen’s *d* = 0.4) scores than SSP children. SPARK children also exhibited significantly higher CBCL total (*t*(103.26) = 5.02, *P* < 0.001, Cohen’s *d* = 0.46), CBCL DSM-5 ADHD (*t*(103.20) = 4.42, *P* < 0.001, Cohen’s *d* = 0.41) and CBCL DSM-5 Anxiety (*t*(103.43) = 3.94, *P* < 0.001, Cohen’s *d* = 0.35) scores, as well as marginally significant CBCL DSM-5 Depression scores (*t*(103.05) = 2.82, *P* = 0.006, Cohen’s *d* = 0.27, not significant after Bonferroni’s correction). SPARK children had lower Vineland Adaptive Behavior Composite (*t*(103.62) = −4.39, *P* < 0.001, Cohen’s *d* = −0.36), Vineland communication (*t*(104.42) = −5.66, *P* < 0.001, Cohen’s *d* = −0.41) and Vineland daily living skills (DLS) (*t*(103.39) = −4.41, *P* < 0.001, Cohen’s *d* = −0.38) scores, as well as marginally significant socialization scores (*t*(102.89) = −2.32, *P* = 0.02, Cohen’s *d* = −0.23, not significant after Bonferroni’s correction).

**Fig. 2:**
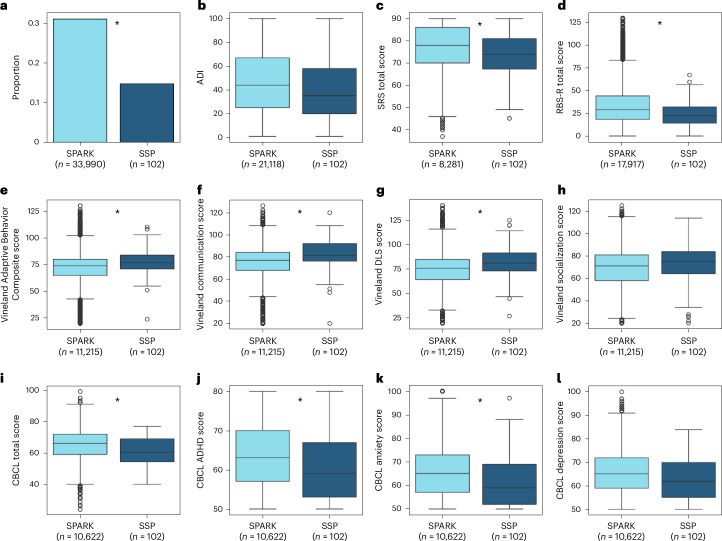
Comparison of autistic children in the SSP (dark blue, 102 participants) versus SPARK without SSP children (light blue, number of participants noted per measure). **a**, Proportion of children with clinically diagnosed sleep problems as reported by parents on a medical background questionnaire (33,824 SPARK participants). **b**, ADI as reported in SPARK data and computed according to the participants’ address using 2019 US Census data (20,952 SPARK participants). **c**, SRS total scores (8,115 SPARK participants). **d**, RBS-R total scores (17,751 SPARK participants). **e**, Vineland Adaptive Behavior Composite scores (11,049 SPARK participants). **f**, Vineland communication scores (11,049 SPARK participants). **g**, Vineland DLS scores (11,049 SPARK participants). **h**, Vineland socialization scores (11,049 SPARK participants). **i**, CBCL total scores (10,456 SPARK participants). **j**, ADHD symptoms from the CBCL (10,456 SPARK participants). **k**, Anxiety symptoms from the CBCL (10,456 SPARK participants). **l**, Depression symptoms from the CBCL (10,456 SPARK participants). The asterisks indicate significant differences between SSP and non-SSP SPARK groups according to two-sided *χ*^2^ (**a**) or Student’s *t*-tests (all other panels), after Bonferroni’s correction for 12 comparisons (*P* < 0.05, corrected). The boxplots present median and interquartile range (IQR) and the whiskers are drawn to the farthest datapoint within 1.5× the IQR of the 25th or 75th percentile, respectively. Participants beyond this range are individually marked (outliers).

Finally, the proportion of parents who reported that their children had clinically diagnosed sleep problems on the SPARK medical background questionnaire was significantly higher in SPARK than in SSP participants (*χ*²(1) = 12.03, *P* < 0.001), whereas socioeconomic status, as estimated by the 2019 national area deprivation index (ADI)^42^, did not differ significantly across the two groups (*t*(88.74) = 1.88, *P* = 0.06, Cohen’s *d* = 0.2). Hence, although the behavioral scores of SSP autistic children clearly fall within the range of SPARK children (Fig. 2), their scores, on average, indicate slightly weaker core autism symptoms, fewer behavioral and mental health challenges, fewer clinically diagnosed sleep problems and slightly better adaptive functioning.

### Behavioral characterization of autism and sibling groups

**Fig. 3:**
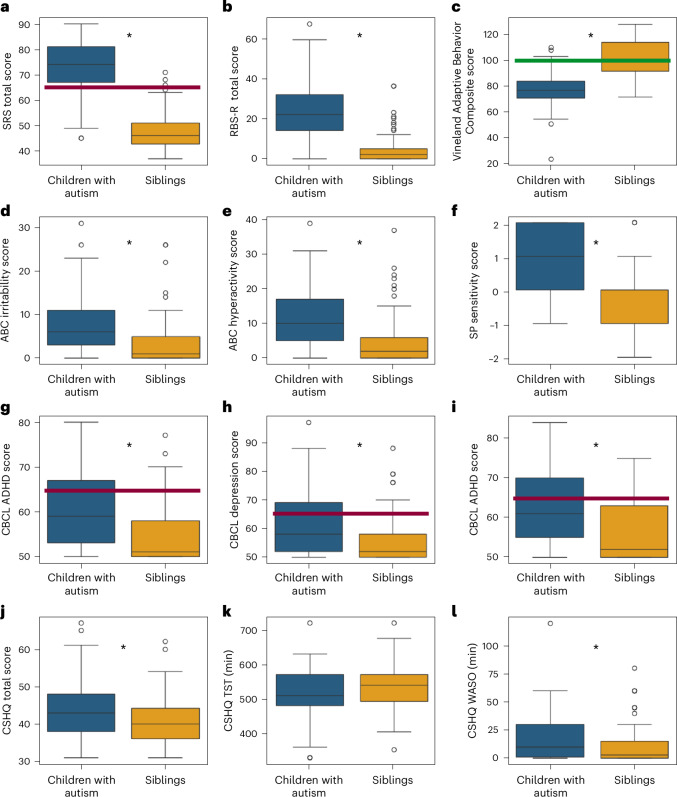
Parent-reported behavioral symptoms and abilities in autistic children (blue, *n* = 102) and siblings (yellow, *n* = 98). **a**, SRS. **b**, RBS-R. **c**, Vineland Adaptive Behavior Composite. **d**, Irritability subscale from the ABC. **e**, Hyperactivity subscale from the ABC. **f**, Sensory sensitivity subscale from the Sensory Profile. **g**, ADHD symptoms from the CBCL. **h**, Anxiety symptoms from the CBCL. **i**, Depression symptoms from the CBCL. **j**, CSHQ total sleep disturbance score. **k**, CSHQ parent-reported TST in minutes. **l**, CSHQ parent-reported WASO in minutes. The red lines indicate the clinical cutoff (*t* score = 65) and the green line the population mean. The asterisks indicate significant differences between autism and sibling groups according to mixed linear model analyses (*P* < 0.05, two sided, uncorrected). The boxplots present median and IQR and the whiskers are drawn to the farthest datapoint within 1.5× the IQR of the 25th or 75th percentile, respectively. Participants beyond this range are individually marked (outliers).

We examined differences across autism and sibling groups in key measures of core autism symptoms, adaptive behaviors, challenging behaviors, sensory sensitivities, co-occurring psychiatric symptoms and sleep behaviors (Fig. 3). We performed a mixed linear model analysis for each measure to assess differences across autism and sibling groups (fixed effect) while accounting for potential differences in parent reports across families (random effects), as well as the age and sex of the participating children (additional fixed effects).

These analyses revealed significantly higher scores in autistic children relative to their siblings on SRS total (*β* = 26.3, *P* < 0.0001, Cohen’s *f*^2^ = 2.75), RBS-R total (*β* = 21.1, *P* < 0.0001, Cohen’s *f*^2^ = 1.06), Sensory Profile sensitivity (*β* = 1.4, *P* < 0.0001, Cohen’s *f*^2^ = 0.7), ABC irritability (*β* = 5.3, *P* < 0.0001, Cohen’s *f*^2^ = 0.33), ABC hyperactivity (*β* = 6.5, *P* < 0.0001, Cohen’s *f*^2^ = 0.33), CBCL ADHD symptoms (*β* = 5.2, *P* < 0.0001, Cohen’s *f*^2^ = 0.16), CBCL anxiety symptoms (*β* = 6.5, *P* < 0.0001, Cohen’s *f*^2^ = 0.23), CBCL depression symptoms (*β* = 6.27, *P* < 0.0001, Cohen’s *f*^2^ = 0.25) and CSHQ total sleep disturbance scores (*β* = 3.08, *P* = 0.0018, Cohen’s *f*^2^ = 0.07), as well as significantly lower scores on the Vineland Adaptive Behavior Composite (*β* = −24.2, *P* < 0.0001, Cohen’s *f*^2^ = 0.88). Parent-reported TST was, on average, 16 min shorter in the autistic children relative to their siblings but did not differ significantly across groups (*β* = −8.42, *P* = 0.29, Cohen’s *f*^2^ = 0.008). Parent-reported WASO was, on average, 8 min longer in the autistic children and did differ significantly across groups (*β* = 7.66, *P* = 0.006, Cohen’s *f*^2^ = 0.05).

There was no significant effect of sex on any of the parent-reported questionnaires or measures (*P* > 0.1) and a significant effect of age only on ABC irritability (*β* = −0.05, *P* = 0.004), ABC hyperactivity (*β* = −0.04, *P* = 0.03) and TST (*β* = −0.56, *P* = 0.0017). Differences between siblings (within family) were considerably larger than differences across families with intraclass correlation coefficients (ICCs) ranging between 0.16 and 0.37.

### Synchronized recordings with multiple devices

EmbracePlus smartwatch data were recorded continuously when participants wore the device, Dreem3 headband data were recorded when participants activated the device before going to sleep, until they turned it off in the morning, and Withings’ mattress sensor data were recorded automatically whenever participants entered their bed. A single Samsung A51 smartphone was used to manage all devices and synchronized data acquisition to a single clock.

**Fig. 4:**
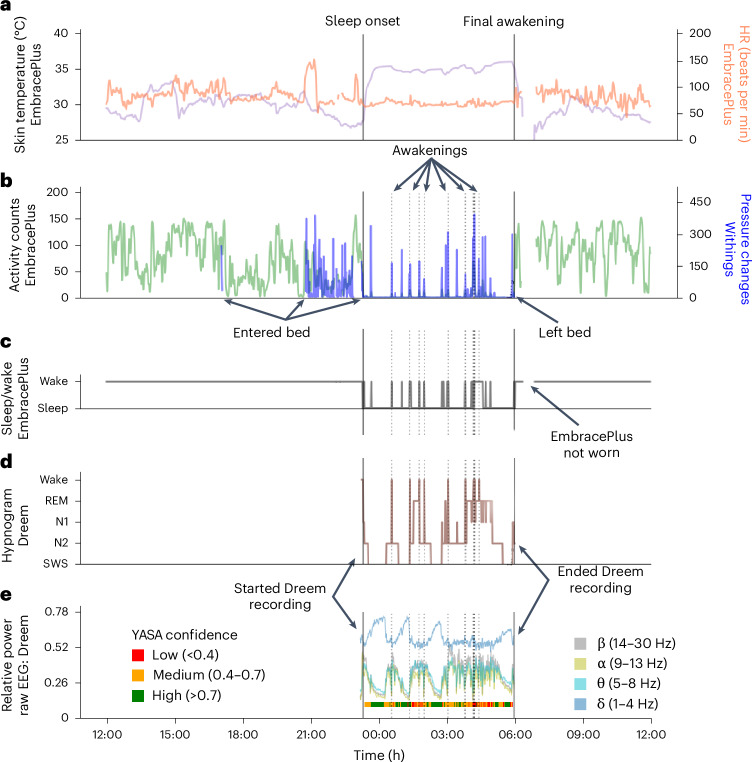
The 24-h recording from a single participant starting and ending at noon. **a**, Skin temperature (purple) and HR (orange). **b,** Activity counts from EmbracePlus accelerometer (green) and pressure changes from the Withings’ sleep mat (blue). **c**, Sleep and wake classification based on accelerometer data analyzed with EmbracePlus algorithms. **d**, Hypnogram from the Dreem3 automatic sleep-staging algorithm. **e**, Average EEG power across all Dreem EEG channels. Average δ (1–4 Hz, blue), θ (5–8 Hz, turquoise), α (9–13 Hz, yellow) and β (14–30 Hz, gray) band power demonstrate multiple sleep cycles throughout the night. The color bar below the EEG power marks segments with high (green), medium (orange) and low (red) sleep staging confidence according to the YASA algorithm, which classifies sleep stages according to EEG features in the time and frequency domains. Classification confidence is likely to depend on EEG data quality. The solid vertical lines mark sleep onset and final awakening as labeled. The dotted vertical lines mark awakenings during the sleep period according to the Dreem algorithm.

This design enabled us to directly compare data across multiple sensor recordings with high temporal fidelity as demonstrated in a 24-h recording from a single participant (Fig. 4). The devices yielded complementary information about the participant’s behavior and sleep or wake state during the night. For example, the activity count time course, which quantifies movements in 1-min epochs using the EmbracePlus accelerometer, was correlated with pressure changes recorded by the Withings’ sleep mat when the participant was in bed (*r* = 0.41, *P* < 0.001; Fig. 4b). Similarly, sleep and wake epochs identified by the Dreem sleep-staging algorithm in the EEG data (Fig. 4c) exhibited partial agreement with sleep and wake epochs identified by the EmbracePlus algorithm using accelerometer data (sensitivity: 0.82; specificity: 0.27; Fig. 4d). Agreement across devices and algorithms varied across participants and nights. Note that the analyses presented here used measures (that is, activity counts and sleep or wake epoch labels) that were generated by the proprietary algorithms of each device manufacturer.

In addition, synchronized raw EEG data are also available, enabling in-depth analysis of nightly recordings with spectral analysis (Fig. 4e). To demonstrate the quality of the EEG data, we applied the Yet Another Spindle Algorithm (YASA) open-source sleep-staging algorithm^43^ to the raw EEG data. YASA classifies 30-s epochs of raw EEG into wake and sleep stages according to time and frequency domain EEG features. The available raw EEG data were of sufficient quality for classifying sleep stages with medium-to-high confidence in the vast majority of epochs (color bar, bottom of Fig. 4e).

### Agreement across devices and with parent-reported sleep measures

**Fig. 5:**
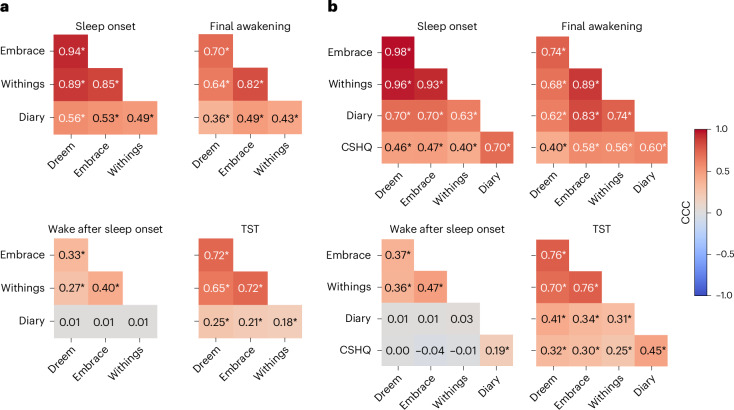
CCCs demonstrating the pairwise agreement across devices, sleep diary and CSHQ in estimating key sleep measures—SO, FA, WASO and TST. **a**, Computed across individual nights. **b**, Computed across participants after averaging measures across nights (per device or diary) and adding CSHQ data. CCCs are coded by color and values are noted for each pair. The asterisks show significant correlation (*P < 0.05, Bonferroni corrected for 50 comparisons, randomization analysis).

We assessed agreement across devices in identifying four basic sleep measures (Fig. 5): sleep onset (SO), final awakening (FA), WASO and TST (time from SO to FA − WASO). We identified nights where all three devices recorded the child’s sleep successfully (Methods) and then extracted SO, FA, WASO and TST as identified by the proprietary algorithm of each device per night. Note that we excluded brief awakenings that were 5 min or shorter when calculating the EmbracePlus WASO estimate to reduce its sensitivity (Methods). The same measures were also extracted from sleep diary data as reported by parents for the same nights. We limited our analyses to children with at least three nights of valid data from all devices and a sleep diary (1,153 nights from 70 autistic children and 67 siblings). This enabled us to also assess agreement between devices and diary measures that were averaged across at least three nights and parent-reported measures from questions in the CSHQ, where parents reported typical, average sleep measures per child.

We computed concordance correlation coefficients (CCCs) to assess agreement between pairs of devices and/or sleep diary measures across nights (Fig. 5a). This analysis demonstrated moderate-to-excellent agreement across the three devices when estimating SO, FA and TST, with weaker yet still significant CCCs for WASO. There were considerably weaker, yet still significant, CCCs between each of the devices and the sleep diary data for SO, FA and TST, but not for WASO, where there was no agreement between the sleep diary and any of the devices.

Next, we performed the same analysis after averaging each measure across all available nights per child (Fig. 5b). This yielded even stronger agreement across devices with moderate-to-excellent CCCs for SO, FA and TST, as well as weaker yet still significant CCCs for WASO. Here too there were considerably weaker, yet still significant, CCCs between each of the devices and sleep diary data for SO, FA and TST, but not for WASO. Adding the CSHQ parent-reported measures revealed moderate CCCs with each of the devices for SO, FA and TST, but not for WASO, where there was no agreement. CCCs between CSHQ and the sleep diary were significant across all measures, including WASO with poor-to-moderate values.

These analyses demonstrated an important key finding: there was far stronger agreement across sleep measures from the three independent devices than there was between any of the devices and parent-reported measures. This finding was particularly strong for WASO, where there was no agreement between devices and parents about the extent of night-time awakenings of individual children whether examining individual nights or their mean per child.

### Comparison of device sleep measures across groups

**Fig. 6:**
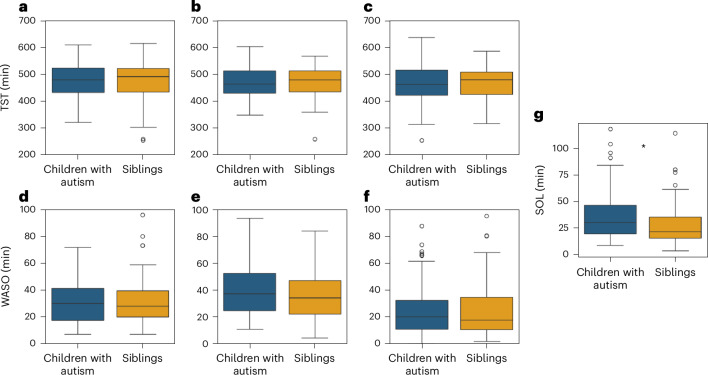
Comparison of TST, WASO and SOL between autistic children (blue, *n* = 70) and their siblings (yellow, *n* = 67). **a**, TST from Dreem. **b**, TST from Embrace. **c**, TST from Withings. **d**, WASO from Dreem. **e**, WASO from Embrace. **f**, WASO from Withings. **g**, SOL from Dreem. The asterisks were significant differences between autism and sibling groups according to a mixed linear model analysis (*P* < 0.05, two sided, uncorrected). The boxplots present median and IQR and the whiskers are drawn to the farthest datapoint within 1.5× the IQR of the 25th or 75th percentile, respectively. Participants beyond this range are individually marked (outliers).

To ensure consistency, we performed all further analyses with the same subset of data described above. We did not find any significant differences between groups in objective TST or WASO measures extracted from any of the three devices (Fig. 6). TST differences between autism and sibling groups were, on average, <2.6 min according to all devices and were not significant (10.4 >*β* > 5.65, *P* > 0.16, Cohen’s *f*^2^ < 0.02). WASO differences between groups were, on average, <4.1 min according to all devices and were also not significant (*β* < 2.3, *P* > 0.53, Cohen’s *f*^2^ < 0.002). It is interesting that the ICCs of TST values from all three devices were between 0.52 and 0.62, demonstrating that much of the variance in sleep duration was explained by differences across families (that is, home environment) rather than differences in diagnosis, age or sex of the children. ICC ratios for WASO values were smaller with a range of 0.001–0.340.

In contrast, SOL differed significantly between groups. We defined SOL as the time from Dreem recording onset to sleep onset, given that families were instructed to start the Dreem recording when the child was ready to go to sleep. We were unable to compute independent SOL measures from the EmbracePlus or Withings’ devices, because we did not have a clear indication of the time that the child was ready to go to sleep from either of these devices. Dreem-defined SOL was, on average, 7.5 min longer in autistic children relative to their siblings, a difference that was statistically significant (*β* = 7.37, *P* = 0.037, Cohen’s *f*^2^ = 0.057). There were no significant effects of sex (*β* = −0.03, *P* = 0.99) or age (*β* = 0.11, *P* = 0.13) on SOL.

### Behavioral difficulties were associated with EEG-derived SOL but not WASO

**Fig. 7:**
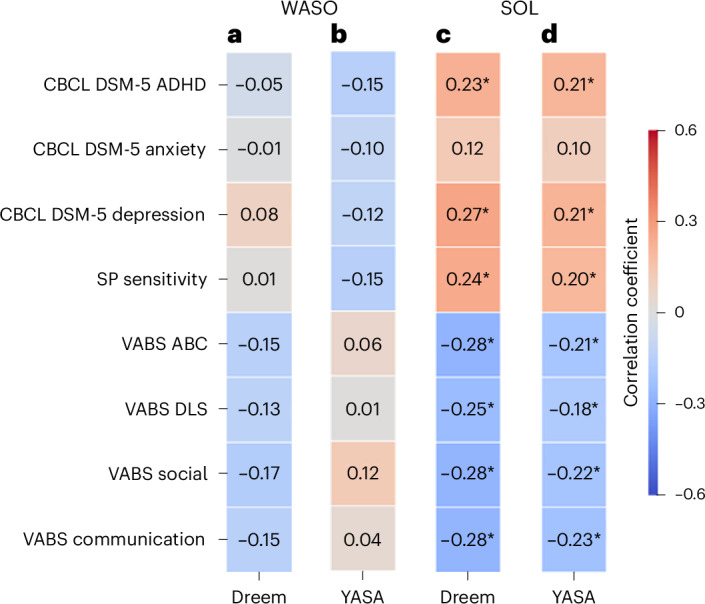
Matrix of Pearson’s correlation coefficients between EEG-defined WASO or SOL measures (columns) and parent-reported behavioral difficulties or abilities (rows). **a**, WASO according to the Dreem algorithm. **b**, WASO according to the YASA algorithm. **c**, SOL according to the Dreem algorithm. **d**, SOL according to the YASA algorithm. The rows correspond to the CBCL, SP and VABS subscale scores. The asterisks indicate significant Pearson’s correlation between the sleep and behavioral measures (*P < 0.05, uncorrected).

Given that EEG recordings enable direct identification of sleep and/or wake epochs and are used as the gold standard for sleep research, we focused our final analysis on EEG estimates of SOL and WASO, which measure difficulties in sleep initiation and maintenance, respectively (Fig. 7). We generated two independent estimates of each sleep measure with the Dreem and YASA^43^ sleep-staging algorithms, which yielded independent hypnograms per night or recording. Note that, in this analysis, we further demonstrate the utility of the raw EEG data available in the SSP, which enabled us to validate the results of the Dreem sleep-staging algorithm with the YASA algorithm^43^, which classifies wake and sleep stages based on EEG features in the time and frequency domains^43^.

In this analysis we adopted a dimensional approach^44^ and examined whether sleep disturbances were associated with behavioral difficulties across study participants, regardless of autism diagnosis. We assessed the relationship between each sleep measure and multiple parent-reported behavioral measures, including CBCL subscales for DSM-5 ADHD, anxiety and depression symptoms (three psychiatric symptom domains that are commonly associated with sleep problems), SP subscale for sensory sensitivity (also commonly associated with sleep problems) and Vineland Adaptive Behavior Scale (VABS) scores that are indicative of general function.

Dreem and YASA estimates of SOL were significantly correlated with all behavioral measures (|*r*| ≥ 0.18, *P* < 0.04) except for CBCL anxiety scores (*r* ≤ 0.12, *P* > 0.14). In contrast, Dreem and YASA estimates of WASO were not significantly correlated (|*r*| ≤ 0.17, *P* > 0.05) with any of the behavioral measures (Fig. 7a,b). Hence, equivalent results were apparent across independent YASA and Dreem estimates. We, therefore, averaged the SOL and/or WASO values across Dreem and YASA to create a single value of each measure per participant and computed their correlation with each of the behavioral measures noted above. We then directly compared the SOL and WASO correlation values using Steiger’s *Z*-tests (Methods). SOL correlations were significantly stronger than WASO correlations (*P* < 0.05) for all behavioral measures except CBCL anxiety and Vineland DLS scores. These results demonstrate a dissociation between EEG-defined SOL and WASO, suggesting that sleep initiation problems, rather than sleep maintenance problems, are significantly associated with a variety of behavioral difficulties and poorer adaptive behaviors, regardless of autism diagnosis.

## Discussion

This study presents the extensive data available in the SSP, which includes raw and processed, high-resolution, synched data from multiple devices and sensors (Figs. 1 and 4) along with parent-reported questionnaires and daily sleep diaries (Fig. 3). These data offer rich, unique opportunities to study sleep behavior and neurophysiology as well as daytime behaviors in autism. The data also offer broad opportunities for methodological development, including training deep learning algorithms to identify and quantify a variety of behaviors and states (for example, hyperactivity). Furthermore, whole-exome sequencing data are also available for all participants and their parents, enabling potential investigations of genotype–phenotype relationships.

An important feature of the SSP is the simultaneous collection of multiple objective and subjective measures of sleep that enable their comparison. Initial analyses of common sleep measures, including SO, FA, WASO and TST, demonstrated considerably larger agreement across device pairs (after reducing EmbracePlus sensitivity to brief awakenings) than between each device and parent-reported sleep diary or CSHQ measures (Fig. 5). Averaging measures across multiple nights per participant increased agreement across devices, demonstrating the value of multi-night recordings for reducing device measurement errors on individual nights. Most importantly, these findings reveal that objective sleep measures, acquired with three independent devices and sensor types, yielded more accurate and reliable data than subjective parent reports. Note that device recordings included sleep EEG data, which is typically used as ‘ground truth’ for classifying sleep and/or wake periods. This motivates further research with multi-device, multi-night study designs that can accurately quantify sleep in the home environment.

Comparison of SSP participants with autism with the entire SPARK cohort demonstrated that these children had slightly more moderate behavioral symptoms and fewer clinically diagnosed sleep problems relative to their age-matched peers with autism in SPARK (Fig. 2). This suggests that data in the SSP sample may somewhat underestimate sleep and behavioral problems in the broad autism population and motivate further data collection from autistic children with more severe symptoms.

Nevertheless, parents reported greater sleep problems in SSP children with autism relative to their siblings, including significantly larger WASO and higher CSHQ total sleep disturbance scores (Fig. 3). When analyzing objective sleep measures from the devices, we found that only SOL was significantly longer in the autism group (Fig. 6). Given the limited agreement between device and parent-reported sleep measures (Fig. 5), this suggests that parent-reported sleep disturbances such as prolonged WASO are not necessarily evident in direct, objective measures of sleep. Indeed, a recent meta-analysis of Actigraphy studies in autism also reported that SOL was significantly longer in autistic children relative to typically developing controls whereas WASO was not^2^. PSG studies have also mostly reported no difference in WASO across groups^21–24^. Although these studies yield converging evidence in agreement with our findings, it is important to note that SOL and WASO are particularly difficult to measure with wearable devices^31,32^. Moreover, in the current study we did not have a direct measure of ‘lights off’, which is important for objectively defining the onset of the SOL period. Instead, our estimates were based on the initiation of the Dreem3 EEG recordings before bedtime.

Given that siblings of autistic children are known to have elevated rates of developmental and behavioral difficulties relative to the general population (see below), we also analyzed the data using a dimensional approach^39,44^. Here we examined whether the severity of common co-occurring psychiatric symptoms (ADHD, anxiety and depression), as estimated by the CBCL, sensory sensitivity, as estimated by the Sensory Profile (SP), and adaptive function, as estimated by the Vineland, were related to two key sleep disturbance measures: WASO and SOL. We performed this analysis independently with two sleep-staging algorithms (Dreem and YASA) and demonstrated equivalent results across both. Although SOL was significantly correlated with multiple behavioral measures, WASO was not (Fig. 7). Moreover, a direct comparison of SOL and WASO correlation values demonstrated significantly stronger correlations for SOL in all but two behavioral measures. This dissociation highlights the need to further study sleep initiation problems and their underlying physiological mechanisms in children with behavioral and developmental difficulties, while using direct, objective sleep measures.

The examples above demonstrate a small subset of potential sleep analyses that can be explored using processed or raw data in the SSP. Further studies could examine additional sleep measures including sleep regularity (that is, across nights), diurnal rhythm, sleep architecture and sleep neurophysiology (for example, sleep spindles) that may hold important insights for basic and clinical autism research.

### The SSP sibling study design

The sibling study design implemented in the SSP is both a feature and a limitation of this resource. Parents reported that participating children who were autistic exhibited significantly greater behavioral difficulties than their nonautistic siblings, with particularly large differences in the severity of core autism symptoms, as expected (Fig. 3). Nevertheless, siblings of children with autism are known to exhibit more elevated rates of developmental and behavioral problems than the general population, including high rates of ADHD, language delays, cognitive difficulties and mental health problems^36,37^. Indeed, parents of children in the SSP reported that 58% of children with autism and 30% of siblings had a medical diagnosis of ADHD (Table 1). Moreover, multiple studies have reported that siblings often have mild autism symptoms that place some of them within the broad autism phenotype^45^.

When studying autism using SSP data, it is, therefore, important to consider that sibling data are not equivalent to ‘control’ data from children free of any developmental concerns, as is common in categorical case–control studies. Lack of significant differences in TST between autism and sibling groups (Fig. 3) should not be interpreted as evidence for lack of sleep disturbances in children with autism. Rather, both autism and sibling groups may exhibit reduced TST in comparison to children without developmental concerns due to high rates of other psychiatric conditions (for example, ADHD), which are also associated with sleep disturbances^4^. The SSP may therefore be particularly useful for studies that adopt a dimensional or transdiagnostic approach, with the goal of unraveling relationships between multiple behavioral and neurophysiological dimensions regardless of categorical diagnoses (for example, Fig. 6).

Although the sibling design may seem to introduce some complexity, it simultaneously affords several advantages. First, it constrains multiple environmental and familial factors that are usually not accounted for in case–control study designs. For example, sleep behavior is strongly influenced by familial genetics^46^ and sleep hygiene^47^. Such factors are likely to differ across autism and unrelated control groups yet are constrained in autism–sibling pairs. Indeed, our mixed-model analyses of all sleep measures included sibling pairs as a random-effects factor and revealed that >50% of the variance in TST values (in all three devices) was explained by between-family differences rather than autism–sibling differences. Second, subjective parental reports were collected for both diagnostic groups from the same parents, thereby reducing between-group differences in reporter bias^48^. These advantages suggest that, when significant differences across autism–sibling pairs are detected in this cohort (for example, SOL; Fig. 5), they are likely to be of high validity given that multiple environmental factors are controlled. A final additional advantage is the availability of whole-exome DNA sequencing data from all participants and their parents, offering the opportunity to use familial genetic techniques to test for potential gene–phenotype relationships, although such research will likely require expanding the number of participants in the SSP to enable higher statistical power.

### The remote at-home SSP study design

Previous autism sleep studies have mostly used questionnaires, Actigraphy devices or PSG recordings that were performed during a single night, mostly in a sleep lab. These studies typically focused on one measurement technique at a time with Actigraphy and PSG studies often recruiting relatively small samples^2,21–25^.

The SSP combines multiple research techniques in a remote study design where wearable devices were mailed to the families who operated them independently at home. This design had several key advantages over previous studies. First, data were collected at home (that is, high ecological validity) rather than in a sleep lab. This is particularly meaningful in autism research, because children with autism often find changes in routines particularly difficult^28^ such that sleep measurements from a sleep laboratory may not represent typical sleep at home. Second, as data were collected with a wearable EEG device rather than a traditional PSG system, we were able to record most participants across multiple days and/or nights. This was important for ensuring that children with autism acclimated to the EEG devices^49^ and critical for allowing future studies to calculate measures of sleep regularity and diurnal rhythm that require multiple nights of data. Third, as the SSP utilized multiple devices in parallel (Fig. 3), it is possible to compare data and demonstrate the reliability of findings across devices (Fig. 4), addressing important concerns about reproducibility^50^. Fourth, remote data collection enabled us to record data from up to 30 families in parallel; performing an equivalent study in a sleep laboratory would have required significantly greater resources. Fifth, all our devices continuously streamed data to the device company servers, enabling us to monitor data acquisition and troubleshoot with families when necessary. Finally, remote data collection can afford families in isolated locations and those in low socioeconomic situations an equal opportunity to participate in research. The current study included families from 22 states throughout the USA, including those living in remote rural areas.

Despite the many advantages listed above, remote studies also introduce a variety of challenges. First, some participants were not able to successfully record multiple nights with all devices. Hence, only a subset of the collected data was used when comparing data across devices (Figs. 5 and 6). Note that future studies examining individual device data or even pairs of devices will be able to work with considerably larger subsets of the SSP data than included in our stringent analyses. Second, as the selected devices are relatively new and have been validated in only a relatively small number of studies^34,51,52^, their measures may not be directly comparable to those of older Actigraphy devices or lab-based PSG systems. Indeed, the Dreem EEG data include only a partial PSG montage with no electro-oculogram, electromyogram or mastoid channels, thereby limiting the analyses that can be performed with these data. Third, devices that can be used remotely are often suitable only for older children and adults. For example, the Dreem headband requires a minimal head circumference of 52 cm and is not likely to fit most children under the age of 10. Hence, some devices implemented in the SSP will not be appropriate for remote studies of younger cohorts. Finally, remote data collection is far less controlled than research in the lab. Participants may inadvertently rotate or remove devices in the middle of the night or use them in unexpected ways when they are not monitored by researchers. Hence, it is important to assess data quality and define clear criteria for exclusion. Initial steps to enforce such criteria in the SSP are exclusion of recordings where TST was <3 h or >16 h, WASO >3 h and/or recordings where EEG was of low quality according to Dreem criteria. Hence, an important principle of remote data collection is to acquire extensive data with the expectation that it will contain anomalies and noise which will require careful cleaning and strict exclusion criteria. Ideally, researchers will formulate open-source processing pipelines for SSP data that can be shared so that additional data-cleaning steps are standardized across future studies.

### Digital phenotyping with wearable or nearable devices: beyond sleep

In the initial analyses of the SSP, we focused on objective quantification of sleep disturbances in autism, a commonly reported co-occurring condition of high priority to individuals with autism and their families. However, the SSP offers a wide variety of data for studying a variety of behaviors beyond sleep. For example, recent studies have utilized EDA recordings to identify tantrums and emotional outbursts in children with autism and challenging behaviors^5^, whereas others have used Actigraphy to quantify hyperactivity in children with ADHD^53^. The availability of raw accelerometer, EDA, skin temperature and PPG data from multi-day EmbracePlus recordings, along with daily parent-reported data on tantrums and emotional regulation, may enable a variety of studies on these topics in autism using the SSP dataset.

## Conclusions

The SSP is a new open-science resource that affords researchers from multiple disciplines a unique opportunity to study human behavior, sleep and autism using digital phenotyping techniques. It demonstrates the feasibility of recording sleep and daily behavior remotely from a relatively large sample of children with and without autism, using multiple synchronized devices in parallel over an extended period. Initial analyses demonstrate the power of this dataset and reveal the specific prominence of sleep initiation problems in autism, as well as their association with a variety of developmental and behavioral problems in both children with autism and their siblings. SSP data, analysis code from this study and whole-exome sequencing data from all participants and their parents are available through SFARI base. We encourage the research community to take advantage of these free, open resources and hope that the SSP will be used widely to advance research in autism and beyond.

## Methods

### Participants

All participating families were recruited from the SPARK cohort^41^, which includes over 150,000 individuals with autism in the USA, many of whom have contributed DNA samples for whole-exome sequencing and completed basic medical history and other questionnaires. In many cases, immediate family members have also joined SPARK, contributed DNA samples and consented to be re-contacted for participation in additional studies. SPARK families were contacted by email with the opportunity to join the SSP if they had two adolescent children who were full siblings, aged 10–17 years, one with a parent-reported medical diagnosis of autism and the other without. In addition, all children had completed whole-exome sequencing, which did not yield any returnable autism-related genetic results (that is, all participants with autism were idiopathic), and all children with autism exceeded the autism cutoff score (>15) on the Social Communication Questionnaire, Lifetime Edition, which was completed by a caregiver at the time that they joined SPARK. All parents completed informed consent and the study was approved by the Western Copernicus Group (WCG) institutional review board committee. Families received US$125 in gift cards for the completion of data collection from each child.

A total of 2,609 SPARK families fulfilling the inclusion criteria described above were contacted by email and 315 families completed the informed consent and were recruited. Of these, 113 families completed all baseline questionnaires for both children and were sent wearable devices. Of these families 11 dropped out of the study for a variety of reasons, including difficulties using the devices, familial difficulties or other challenges. A total of 102 families living in 22 states throughout the USA successfully completed the study with data recorded from 102 children with autism and 98 siblings (Table 1).

### Procedure

Recruited families completed nine parent questionnaires for each of their children before receiving the devices (Table 1). These included the CSHQ^54^, Vineland Adaptive Behavior Scales^55^, CBCL^56^, ABC^57^, SRS-2^58^, RBS-R^59^, Sensory Profile (SP), 2nd edn^60^, Family Inventory of Sleep Habits (FISH)^61^ and a short medical update questionnaire in which parents were asked to report on medically diagnosed conditions (beyond autism) for each of their children (Supplementary Table 1).

Families were then sent a package with the Dreem3 EEG headband (Beacon Inc.), EmbracePlus smartwatch (Empatica Inc.), Withings’ sleep mat (Withings Inc.), a Samsung A51 smartphone set up with apps for the three devices to enable continuous data transfer and written instructions on how to use all the devices. Study coordinators completed an onboarding procedure with each family, ensuring that data collection began with the child who is autistic and instructing one of the parents to complete a daily sleep diary online, using a link from a daily text message reminder. Parents also reported on medications taken during the study as part of the daily sleep diary (Supplementary Table 2). We grouped medications according to their clinical indication (Supplementary Table 3) when presenting results across autism and sibling groups (Table 1).

Data collection was monitored remotely and families were contacted when data were missing or of low quality. After approximately 3 weeks of data collection from the child with autism, devices were transferred to their sibling and data collection continued for another 3 weeks. We prioritized data collection from children with autism, who were always recorded first, because these data were more valuable for the purposes of the study. Finally, the devices were returned by courier and the families were given a gift card and sent a sleep report summarizing some of the data collected from each of their children.

### Data acquisition and structure

The Dreem3 headband recorded EEG data from five channels over the frontal and occipital cortex (F1, Fz, F2, O1 and O2) at 250 Hz, as well as accelerometer data at 50 Hz. Participants were instructed to turn on and wear the device before going to bed and turn it off when they woke up in the morning. Data were uploaded automatically to Beacon Inc. servers every time the headband was charged. Their automated system then applied a bandpass filter with a low cutoff frequency of 0.4 Hz and a high cutoff frequency of 35 Hz, generating a single H5 and EDF file with the raw EEG per night. In addition, the Beacon system also automatically generated a hypnogram file per night in txt format using their proprietary sleep-staging algorithm and a summary statistics file with common sleep measures such as TST and WASO per night. Recordings containing <3 h or >16 h of TST were discarded from the SSP and are not reported in this article. Further details about Dreem3 data acquisition and structure are available on their website (https://beacon.bio/dreem-headband).

The EmbracePlus smartwatch recorded data from four sensors: accelerometer (at 64 Hz), PPG (at 64 Hz), EDA (at 4 Hz) and skin temperature (at 1 Hz). Raw data were continuously transmitted from the device to the Care Lab app on the cellphone via Bluetooth and then automatically transferred and stored on the Empatica servers in JSON files of variable lengths. We concatenated JSON files with raw data from each sensor into a single CSV file per 24 h per sensor, starting at 12am according to their timestamps. Segments without data (for example, participants took off the watch) were replaced with not-a-number values to maintain equivalent file structure. In addition, Empatica automatically computes summary statistics of the data which include, for example, step-count, HR, HR variability and sleep–wake classification per minute. These data were also reformatted into CSV files with the same 24-h structure as the raw data. Further details about EmbracePlus data acquisition and structure are available on their website (https://www.empatica.com/embraceplus).

The Withings’ sleep mat contains an inflated chamber that is placed under the mattress in the area corresponding to the participant’s torso. Raw data are not available from the device, but pressure changes, HR, movement statistics and sleep–wake classification measures are available per minute (0.016 Hz). These data are recorded only when the participant is in bed and stored in a separate CSV file per night. Recordings of <3 h or >16 h of TST were discarded from the SSP and are not reported. Further details about Withings’ data acquisition and structure are available on their website (https://www.withings.com/us/en/sleep).

Finally, a designated parent received SMS text messages every morning and evening with six to eight questions about the child’s behavior during the preceding night and day, respectively, as well as a question about medication intake (Supplementary Tables 2 and 3). A sleep diary was successfully collected for 188 of the participants (autistic = 95, siblings = 93).

### Data harmonization

Harmonization of data across the three devices and sleep diary involved several steps. First, collected data, initially organized by device user IDs, was mapped to SPARK participant IDs. Second, data timestamps were adjusted to the participants’ local time zone. Third, nightly Dreem and Withings’ recordings were labeled according to the date of final awakening and recordings with <3 h or >16 h of TST were excluded. Most wearable device studies use similar criteria to identify the main sleep period per night before including data in their analyses^62–64^. Most importantly, we used the same criteria across all devices. Fourth, EmbracePlus raw JSON data files of varying lengths were concatenated and split into separate files containing 24 h of data. All data were transformed into equivalent CSV format, except for raw EEG data that were kept in EDF format. Additional information about filenames, data format and data structure are available when accessing the data repository.

### SPARK data

The July 2024 data release of SPARK phenotypic data was downloaded from SFARI Base and analyzed in the current study. This release included data from the following questionnaires: background history, medical history, CBCL, SRS-2, RBS-R and Vineland and Social Communication Questionnaire (SCQ). In addition, ADI percentiles, based on participants’ addresses according to the 2019 US Census Bureau ranking^42^, were also available. We extracted the available data of all 10-year-old to 17-year-old individuals with autism, excluded SSP children and compared the scores of SPARK and SSP participants.

### Data analysis

We first quantified the total number of nights recorded with each device and their overlap (Fig. 1). In this analysis we identified sleep periods in the EmbracePlus data as described below (that is, identified SO, FA, TST and WASO per night) and then counted the number of nights with at least 3 h of TST per device. Next, we compared questionnaire data across SSP and non-SSP SPARK samples (Fig. 2), as well as across autism and sibling groups in the SSP (Fig. 3). To demonstrate the available data, we selected a representative participant and present selected measures from the three devices during a 24-h period (Fig. 3).

All further analyses included data from 70 autistic children and 67 siblings who successfully recorded at least three valid nights with all devices and a sleep diary. A valid night recording included a TST of 3–16 h that started between 7pm and 7am and WASO that did not exceed 3 h from each of the devices. All nights that did not fulfill these criteria were excluded with the assumption that they contained extreme values that were likely caused by device failures (for example, running out of power) or algorithm errors. In addition, Dreem recordings with a quality score of ≤30 out of 100 (as computed by Beacon) were also removed. Using these criteria, we isolated 1,153 nights with high-quality simultaneous data recordings from all three devices and the sleep diary.

Dreem and Withings’ algorithms yield estimates of SO, FA, WASO and TST as part of their nightly reports. EmbracePlus algorithms, however, only yield sleep and wake labels per 1-min epoch of recording, rather than overall nightly reports. We calculated SO, FA, WASO and TST from the EmbracePlus data using the following criteria: SO was defined as the first sleep epoch followed by at least 10 min of consistent sleep; FA was defined as the first wake epoch after the last 10-min segment of sleep that was followed by at least 2 h awake; WASO was calculated as the sum of wake epochs between SO and FA, after excluding all wake segments ≤5 min consecutively. We excluded these brief awakenings throughout the night, because the EmbracePlus sleep and wake classification algorithm seemed to be oversensitive to awakenings and initially yielded WASO estimates that were twice as high as the other two devices, including an excessive number of brief awakenings in all participants. We selected the 5-min threshold because it is commonly used for differentiating brief unnoticed awakenings from those that are remembered and of larger clinical concern^65,66^ and it yielded, on average, similar WASO estimates to those of the other devices (Fig. 6). TST was calculated as the time between SO and FA − WASO. SOL was computed for Dreem data as the time between recording start and SO.

Parents reported SO, FA and WASO per night as part of the sleep diary, enabling us to compute daily TST (TST = FA − SO − WASO). Parents also reported SO, FA and WASO on the CSHQ, but, although SO and FA were reported separately for weekdays and weekends, WASO was reported regardless of day type. We, therefore, computed the mean SO or FA across weekdays and weekends before computing TST.

In our final analysis, we also extracted independent SO, FA, WASO and TST values from hypnograms that were computed from the raw EEG recordings using the YASA sleep-staging algorithm^43^. We applied the YASA algorithm to raw EEG data from all available channels in each EDF file (that is, night). YASA yields a sleep stage score per 30-s epoch per channel, indicating its confidence in identifying the proper sleep stage within a given epoch. We identified a single sleep stage per epoch by computing the majority vote across all channels, thereby yielding a hypnogram per night. SO was defined as the first epoch in the first 10-min segment of sleep. FA was defined as the first wake epoch after the last 10-min segment of sleep that was followed by the end of the recording or 2 h of wake. TST was calculated as the time between SO and FA, excluding WASO. WASO was calculated as the sum of wake epochs between SO and FA.

SOL was computed for Dreem data as the time from the start of recording to SO according to the Dreem algorithm. Note that families were instructed to start the Dreem recordings when the child was ready to go to sleep. SOL was also computed for YASA data as the time from start of recording to SO according to the YASA algorithm. Nights with SOL values exceeding 120 min were considered outliers and excluded from all analyses.

### Statistical analysis

All statistical analyses were performed using custom written Python code. Comparison of questionnaire scores across SSP and SPARK participants with autism was performed with Student’s *t*-tests assuming unequal variance and using Bonferroni’s correction for multiple comparisons and Cohen’s *d* for estimating effect sizes. Proportions of parent-reported clinical sleep problems were compared using a *χ*^2^ test. Comparison of questionnaire scores and sleep measures across autism and sibling groups was performed with mixed linear model analyses as implemented in the Statsmodel Python package. We used family ID as a random effect and diagnosis (autism or sibling), age and sex as fixed effects. This enabled us to separate potential differences across families that are likely to affect both siblings living in the same household from individual effects of diagnosis, age and sex. We also computed ICCs (that is, between-family variance divided by the sum of between-family and within-family variance) to determine whether differences between families were smaller or larger than differences across individuals. The effect size of the diagnosis predictor was estimated with Cohen’s *f*^2^ measure which quantifies the added amount of variance explained by the mixed linear model when adding the predictor.

We computed CCCs to assess pairwise agreement across sleep measures from the different devices, sleep diary and sleep questionnaire (CSHQ). CCC is a common measure of reproducibility where error is calculated as the distance from the diagonal (absolute agreement) rather than from a linear fit line (relative agreement). To determine the significance of CCCs, we performed a randomization analysis where we created a distribution of CCCs expected by chance when shuffling values across nights (that is, shuffling date labels) in 5,000 different iterations. For a CCC to be considered significant, it had to fall beyond the 0.1th top percentile of this distribution (equivalent to a *P* value of 0.001). We chose this conservative *P* value to correct for multiple comparisons, across devices, sleep diary and CSHQ, performed for each sleep measure.

Finally, we performed Pearson’s correlation analyses to assess the relationship between parent questionnaire scores and WASO or SOL measures, as extracted from Dreem recordings with the Dreem or YASA algorithms. Statistical significance was set to a *P* value of 0.05 and we did not correct for multiple comparisons in this analysis to maintain sensitivity. Note that the goal of this analysis was to determine whether consistent relationships with behavior were apparent for either sleep measure across the two sleep-staging algorithms. We then computed the mean WASO/SOL values across Dreem and YASA estimates to yield a single WASO/SOL measure per participant and again computed their Pearson’s correlation with the same behavioral questionnaire scores. Finally, we directly compared SOL and WASO correlation coefficients using Steiger’s *Z*-test for dependent correlations to determine whether SOL correlations were significantly different from WASO correlations.

In all statistical tests, data distribution was assumed to be normal but this was not formally tested. In addition, data collection and analysis were not performed blind to the conditions of the experiments.

### Reporting summary

Further information on research design is available in the Nature Portfolio Reporting Summary linked to this article.

## Online content

Any methods, additional references, Nature Portfolio reporting summaries, source data, extended data, supplementary information, acknowledgements, peer review information; details of author contributions and competing interests; and statements of data and code availability are available at 10.1038/s41593-025-02146-3.

## Supplementary information

Supplementary Information Supplementary Tables 1–3.

Reporting Summary

## Data Availability

SSP data are available through SFARI Base at https://base.sfari.org/dataset/DS0000089. To gain access, validated researchers from academic institutions must submit their local institutional review board approval and agree to the SFARI data transfer agreement. Related whole-exome sequences for all participants are also available through SFARI Base by requesting the SPARK dataset (https://www.sfari.org/resource/spark/). All processed data presented in the results and figures of the current paper are available at https://github.com/Dinstein-Lab/SSP_manuscript.

## Code availability

Python code used to analyze the data and generate the figures is available at https://github.com/Dinstein-Lab/SSP_manuscript.
