# Supplementary material for: An open science resource for accelerating scalable digital health research in autism and other neurodevelopmental conditions
Source: Nat Neurosci. 2025 Dec 30;29(2):467–78. doi: 10.1038/s41593-025-02146-3 (PMC12880914; doi:10.1038/s41593-025-02146-3)
Supplement: Supplementary file 1 — Supplementary Tables 1–3. [file 41593_2025_2146_MOESM1_ESM.pdf]

nature neuroscience

Supplementary information https://doi.org/10.1038/s41593-025-02146-3

An open science resource for accelerating scalable digital health research in autism and other neurodevelopmental conditions

In the format provided by the authors and unedited

An open science resource for accelerating scalable digital health research in autism and other neurodevelopmental conditions – supplementary materials

Micha Hacohen[1,2], Adam Levy[3], Hadas Kaiser[3], LeeAnne Green Snyder[3], Alpha Amatya[3], Brigitta B. Gundersen[3], John E. Spiro[3], Ilan Dinstein[1,2]

1. Psychology Department, Ben Gurion University of the Negev, Beer Sheva, Israel
2. Azrieli National Centre for Autism and Neurodevelopment Research, Ben Gurion University of the Negev, Beer Sheva, Israel
3. Simons Foundation, New York, NY, USA

* Corresponding author, dinshi@bgu.ac.il

Supplementary Table 1: Medical update questionnaire. Participants were able to select multiple options in response to each question.

1.	**Has your child been diagnosed with any of the following sleep disorders by a physician?** (Please select all that apply)
	• None
	• Insomnia
	• Sleep Apnea
	• Sleep Arousal
	• Nightmare Disorder
	• Sleep Behavior Disorder
	• Hypersomnolence Disorder
	• Narcolepsy
	• Restless Legs Syndrome
	• Circadian Rhythm Sleep Wake Disorders
2.	**Has your child been diagnosed with any of the following behavioral disorders by a physician?** (Please select all that apply)
	• None
	• ADHD/ADD
	• Conduct Disorder
	• Intermittent Explosive Disorder
	• Oppositional Defiant Disorder
	• Repeating Bowel Accidents
3.	**Has your child been diagnosed with any of the following neurological disorders by a physician?** (Please select all that apply)
	• None
	• Brain Infection
	• Seizures Epilepsy
	• Traumatic Brain Injury
	• Tourette Syndrome
4.	**Has your child been diagnosed with any of the following psychiatric disorders by a physician?** (Please select all that apply)
	• None
	• Eating Disorder
	• Alcohol or Substance Use

• Personality Disorder
• Anxiety Disorder
• Bipolar Disorder
• Depression
• Dysregulation Disorder
• Hoarding
• Obsessive Compulsive Disorder

Supplementary Table 2: Sleep diary questions. Evening diary questions were used to report daytime behaviors. Morning diary questions were used to report nighttime behaviors.

Section	Question	Response options
**Evening Diary (PM)**	How energetic was your child today?	**Scale 0-10** (Very tired to Highly energetic)
	How was your child's mood today?	**Scale 0-10** (Terrible to Great)
	How did your child feel physically today?	**1**=Great, **2**=Average, **3**=Minor aches/pains, **4**=Didn't feel well, **5**=Was sick
	Did they have a fever?	**Yes/No**
	Any Gastro-intestinal problems?	**Yes/No**
	Did your child have any tantrums today?	**Yes/No**
	**If yes:** How many tantrums?	**Number** (1-10)
	My child took these medications today	**Free text:** medication name and dosage
	My child consumed caffeinated items in the:	**Multiple choice**: Morning, Afternoon, Evening, None
	My child exercised for at least 20 minutes in the:	**Multiple choice**: Morning, Afternoon, Evening, None
	My child took a nap today?	**Yes/No**
	**If yes:** Nap time	**Time of day** Hour:Minute (AM/PM)
	**If yes:** Nap duration	**Time** Drop-down (15-minute intervals)
**Morning Questions (AM)**	My child got into bed last night at:	**Time of day** Hour:Minute (AM/PM)
	How anxious/restless was your child at bedtime?	**Scale 0-10** (calm/relaxed to anxious/restless)
	I noticed that my child woke up during the night (X) times	Numerical value
	Total minutes awake during night	**Time** Numerical value (Minutes)
	When your child woke up during the night, they:	**Multiple choice**: Left bed, Awakened parents, Required help, Not applicable
	What was the reason for waking up?	**Multiple choice**: Noise, Light, Bad dream, Bathroom, Agitated/anxious/emotional, Not applicable
	This morning my child woke up at:	**Time of day** Hour:Minute (AM/PM)
	When my child woke up for the day, he/she felt:	**Scale 0-10** (Rested to Tired)

Supplementary Table 3: Parent reported medications. Parents reported the use of medication brand names in the evening sleep diary (Supplementary Table 2). These were converted into categorical medication groups as follows.

Category	Medication Brand Name		
**Antidepressants**	Prozac (fluoxetine)<br><br>Zoloft (sertraline)<br><br>Lexapro (escitalopram)	Trazodone<br><br>Cymbalta (duloxetine)<br><br>Wellbutrin (bupropion)	Amitriptyline<br><br>Venlafaxine<br><br>Imipramine
**Stimulants**	Adderall<br><br>Vyvanse<br><br>Focalin (dexmethylphenidate)<br><br>Ritalin (methylphenidate)	Dextroamphetamine<br><br>Jornay<br><br>Concerta	Quillivant<br><br>Azstarys<br><br>Lisdexamfetamine
**Anti-anxiety**	Hydroxyzine<br><br>Buspar (buspirone)<br><br>Ativan (lorazepam)<br><br>Clonidine	Strattera (atomoxetine)<br><br>Depakote<br><br>Guanfacine<br><br>Intuniv	Oxcarbazepine<br><br>Lamictal (lamotrigine)<br><br>Tegretol (carbamazepine)
**Anticonvulsants**	Trileptal<br><br>Depakote	Lamotrigine<br><br>Carbamazepine	Oxcarbazepine
**Antipsychotics**	Abilify (aripiprazole)<br><br>Risperidone (Risperdal)	Seroquel (quetiapine)<br><br>Olanzapine (Zyprexa)	Vraylar<br><br>Ziprasidone
**Sleep Aids**	Melatonin		
