# Supplementary material for: An open science resource for accelerating scalable digital health research in autism and other neurodevelopmental conditions
Source: Nat Neurosci. 2025 Dec 30;29(2):467–78. doi: 10.1038/s41593-025-02146-3 (PMC12880914; doi:10.1038/s41593-025-02146-3)
Supplement: Supplementary file 2 — Reporting Summary [file 41593_2025_2146_MOESM2_ESM.pdf]

nature portfolio

Corresponding author(s): Dinstein

Last updated by author(s): Apr 11, 2025

Reporting Summary

Nature Portfolio wishes to improve the reproducibility of the work that we publish. This form provides structure for consistency and transparency in reporting. For further information on Nature Portfolio policies, see our Editorial Policies and the Editorial Policy Checklist.

Statistics

For all statistical analyses, confirm that the following items are present in the figure legend, table legend, main text, or Methods section.

n/a	Confirmed	
☐	☒	The exact sample size (*n*) for each experimental group/condition, given as a discrete number and unit of measurement
☐	☒	A statement on whether measurements were taken from distinct samples or whether the same sample was measured repeatedly
☐	☒	The statistical test(s) used AND whether they are one- or two-sided<br>*Only common tests should be described solely by name; describe more complex techniques in the Methods section.*
☐	☒	A description of all covariates tested
☐	☒	A description of any assumptions or corrections, such as tests of normality and adjustment for multiple comparisons
☐	☒	A full description of the statistical parameters including central tendency (e.g. means) or other basic estimates (e.g. regression coefficient) AND variation (e.g. standard deviation) or associated estimates of uncertainty (e.g. confidence intervals)
☐	☒	For null hypothesis testing, the test statistic (e.g. *F*, *t*, *r*) with confidence intervals, effect sizes, degrees of freedom and *P* value noted<br>*Give P values as exact values whenever suitable.*
☒	☐	For Bayesian analysis, information on the choice of priors and Markov chain Monte Carlo settings
☐	☒	For hierarchical and complex designs, identification of the appropriate level for tests and full reporting of outcomes
☐	☒	Estimates of effect sizes (e.g. Cohen's *d*, Pearson's *r*), indicating how they were calculated

Our web collection on statistics for biologists contains articles on many of the points above.

Software and code

Policy information about availability of computer code

Data collection	The data was collected using three commercial devices that are described in the manuscript. Links to the technical manuals of the devices are provided in the methods section.
Data analysis	All data analyses were performed with custom written code in Python, which is available as part of a Github repository (link provided in the manuscript)

For manuscripts utilizing custom algorithms or software that are central to the research but not yet described in published literature, software must be made available to editors and reviewers. We strongly encourage code deposition in a community repository (e.g. GitHub). See the Nature Portfolio guidelines for submitting code & software for further information.

Data

Policy information about availability of data

All manuscripts must include a data availability statement. This statement should provide the following information, where applicable:

- Accession codes, unique identifiers, or web links for publicly available datasets
- A description of any restrictions on data availability
- For clinical datasets or third party data, please ensure that the statement adheres to our policy

Clear instructions on how to access the data through SFARI Base are available in the methods section.

Research involving human participants, their data, or biological material

Policy information about studies with human participants or human data. See also policy information about sex, gender (identity/presentation), and sexual orientation and race, ethnicity and racism.

Reporting on sex and gender	We only addressed sex differences in this project as we do not have information about gender. All analyses included sex as a covariate and results are reported accordingly.
Reporting on race, ethnicity, or other socially relevant groupings	We did not examine race, ethnicity, or other social factors in our analyses. Given the relatively small number of families in the study and the familial design (comparison across siblings), we do not think that stratification by these factors is relevant to the reported results and conclusions.
Population characteristics	All of these details are clearly described in the manuscript and available as part of the dataset
Recruitment	Recruitment is clearly described in the manuscript. In short, families who had previously participated in research as part of the SPARK cohort were contacted by email and had the opportunity to participate in the current study. There is likely to be a self selection bias here - for example families who find it difficult to use wearable devices were probably less likely to volunteer for this study.
Ethics oversight	The study was approved by Western IRB

Note that full information on the approval of the study protocol must also be provided in the manuscript.

Field-specific reporting

Please select the one below that is the best fit for your research. If you are not sure, read the appropriate sections before making your selection.

☐ Life sciences ☒ Behavioural & social sciences ☐ Ecological, evolutionary & environmental sciences

For a reference copy of the document with all sections, see nature.com/documents/nr-reporting-summary-flat.pdf

Behavioural & social sciences study design

All studies must disclose on these points even when the disclosure is negative.

Study description	The study includes analyses of quantitative data from multiple wearable devices and parent questionnaires
Research sample	102 families who volunteered for this study - all recruited from the SPARK cohort
Sampling strategy	This study reports data from a sample of convenience. Given the novelty of the data and exploratory nature of the study, we did not perform formal power analyses before data collection. However, all existing Polysomnography and actigraphy studies on autism include smaller samples than that reported in the study. The current sample is, therefore likely to yield higher statistical power than any previously available sample.
Data collection	All data was collected remotely in participants homes as described in the methods. All participants received the same instructions and were able to contact the research team if problems arose with any of the devices.
Timing	Data was collected over a 10 month period between October 2023 and July 2024
Data exclusions	Data was excluded from analyses based on clearly defined measures of quality that are described in the manuscript. In short, participants with fewer that 3 nights of data were excluded from analysis (not from the repository) as we wanted to characterize mean sleep measures across multiple nights.
Non-participation	This is clearly reported in the paper - 11 families dropped out of the study (did not completed data collection)
Randomization	ASD children were compared to their non-autistic siblings. Randomization is not relevant for our design.

Reporting for specific materials, systems and methods

We require information from authors about some types of materials, experimental systems and methods used in many studies. Here, indicate whether each material, system or method listed is relevant to your study. If you are not sure if a list item applies to your research, read the appropriate section before selecting a response.

Materials & experimental systems

n/a	Involved in the study
☒	☐ Antibodies
☒	☐ Eukaryotic cell lines
☒	☐ Palaeontology and archaeology
☒	☐ Animals and other organisms
☐	☒ Clinical data
☒	☐ Dual use research of concern
☒	☐ Plants

Methods

n/a	Involved in the study
☒	☐ ChIP-seq
☒	☐ Flow cytometry
☒	☐ MRI-based neuroimaging

Clinical data

Policy information about clinical studies

All manuscripts should comply with the ICMJE guidelines for publication of clinical research and a completed CONSORT checklist must be included with all submissions.

Clinical trial registration	This is not a clinical trial
Study protocol	This is not a clinical trial - study protocol is described in the methods section
Data collection	All described in the methods section. Data collection was performed remotely in the participants homes.
Outcomes	There were no primary or secondary outcomes - this was not a clinical trial.

Plants

Seed stocks	N/A
Novel plant genotypes	N/A
Authentication	N/A
